# Accumulative experiences of recurrent vulvovaginal thrush: a qualitative study of primary care navigation from patient and clinician perspectives

**DOI:** 10.3399/BJGP.2025.0531

**Published:** 2026-06-02

**Authors:** Tori Ford, Sarah Tonkin-Crine, Gail Hayward, Sue Ziebland, Abigail McNiven

**Affiliations:** 1 Nuffield Department of Primary Care Health Sciences, Radcliffe Primary Care Building, Radcliffe Observatory Quarter, Oxford, UK

**Keywords:** general practice, sexual health, primary health care, patient experience, recurrent vulvovaginal candidiasis, recurrent thrush

## Abstract

**Background:**

Existing research on recurrent vulvovaginal thrush primarily frames experiences through the lens of acute, episodic, and one-off cases of thrush. Studies are lacking that investigate the implications of embedding recurrent cases of thrush into acute frameworks. This paper explores how a condition that is usually seen as one-off transitions into something for patients and healthcare professionals to think about and act on as needing longer-term care.

**Aim:**

To understand patient and clinician perspectives on seeking and providing care for recurrent vulvovaginal thrush, and how these insights might improve healthcare experiences.

**Design and setting:**

This was a qualitative study of patient experiences with recurrent vulvovaginal thrush and healthcare professional perspectives about providing care across England.

**Method:**

Interviews were conducted with 32 patients and 25 healthcare professionals working in primary care and sexual health services in England. Data were analysed using reflexive thematic analysis. Patient and public involvement informed study development and interpretation of results.

**Results:**

Patients and healthcare professionals agreed that acute, transient, and one-off cases of thrush could be self-managed effectively through pharmacy care. When thrush returned, persisted, or evolved, care needed to transition to a different approach, plan, and/or pathway, however, integrating acute episodes could be complex. The themes highlight areas where the needs of people with recurrent vulvovaginal thrush diverged from acute cases, in terms of: a) navigating disjointed health services, b) recognising and responding to recurrence, and c) building ongoing healthcare relationships.

**Conclusion:**

Recurrent vulvovaginal thrush can be managed effectively in primary care, but requires approaches attentive to transitions, collaboration, and recognition of accumulative experiences.

**How this fits in**

Literature highlights that recurrent vulvovaginal thrush has a significant impact on patient lives and that they experience dissatisfaction with existing care pathways. Existing models often frame recurrent thrush through the lens of acute cases of thrush, overlooking vital differences between these. Primary healthcare professionals play a crucial role in providing care for recurrent thrush, but are working with fragmented and disconnected care systems that make pathway transitions challenging. In combining patient and healthcare professional perspectives, this study shows how recurrent vulvovaginal thrush requires distinct approaches from acute cases of the condition.

## Introduction

Vulvovaginal candidiasis, colloquially known as thrush, is a common condition that causes vulval and vaginal itching, burning, and changes in discharge. Most cases are acute, transient, one-off, and easily managed in pharmacy care. However, for 6% of people assigned female at birth, thrush becomes a repetitive, enduring, and long-term condition.^1^ Recurrent vulvovaginal thrush is defined as four or more episodes of thrush within a year.^1^ As such, it typically entails additional or longer engagement with health services.

Past studies report patient dissatisfaction with healthcare experiences for recurrent thrush. Patients perceived general practitioners as having limited interest in recurrent thrush, leading to a ‘loss of confidence’, exacerbated by brief appointments and strained patient–practitioner relationships.^2–4^ Limitations in current diagnostics exacerbate the challenge of recognising and documenting recurrent thrush, leading to differing expectations and perceived missed opportunities .^5^

Care-seeking experiences for conditions that affect women and people assigned female at birth are increasingly framed through a lens of gender health inequity and perceived dismissal.^6^ The Women’s Health Strategy for England reported four in five women had felt they were not listened to or believed by a healthcare professional.^7^ Many women reported that their pain had been labelled as ‘normal’ or ‘natural’.^8^ The Cumberlege report (2020) found that healthcare systems for women’s health are ‘disjointed, siloed, and unresponsive’.^9^ Yet, policy reports have not yet directly considered recurrent thrush.

Our previously conducted systematic review identified a tendency for papers on vulvovaginal thrush to conflate care-seeking experiences for acute and recurrent thrush.^10^ Past work has largely framed recurrent thrush as an additive experience of repeated acute episodes, instead of an ongoing and evolving condition deserving its own consideration. Studies are lacking that investigate the implications of this approach and whether patients and healthcare professionals might experience recurrent thrush management as distinct. Research has yet to fully examine how recurrent thrush care pathways become cyclical, accumulative, or expansive as patients’ behaviours and perceptions relate to past decisions, outcomes, and experiences^10^ — a gap this paper seeks to address.

Existing attempts to map a care pathway for recurrent thrush appear linear and straightforward, while being subsumed or footnoted within frameworks built for acute experiences. For instance, Theroux (2002) interviewed women with vaginal symptoms including recurrent thrush and presents a model around self-treatment.^11^ The model begins with ‘noticing vaginal symptoms’ that leads to ‘making sense of symptoms’ and ‘choosing a treatment path’. Theroux notes that many people choose to ‘bypass the middleman’ and self-treat; which can lead to the relief of symptoms (one possible end to the pathway) or no relief, which returns the patient back to the start at the ‘making sense of symptoms’ stage. Theroux’s pathway does not explore what happens if a patient chooses to consult a healthcare professional.

Providing insight into clinical care-seeking experiences, Donders *et al* (2022) created a model based on a systematic review of vulvovaginal thrush, illustrating steps taken after a healthcare professional is consulted.^12^ This model begins with patients either choosing to self-treat (one possible endpoint) or see a healthcare professional that opens up multiple routes. These options include a misdiagnosis (sending patients back to the start of the model) or a ‘proper diagnosis’. The proper diagnosis is either identified as not vulvovaginal candidiasis (another endpoint), or as vulvovaginal candidiasis, which is then divided into either acute thrush or recurrent thrush (with induction and maintenance treatment, compliance checking, and discussion of pregnancy plans). This model does not show how patient experiences change when they repeatedly interact with this healthcare pathway over time.

Existing studies on healthcare encounters for recurrent thrush rarely enquire into clinician perspectives, or bring them together with patient experiences. Therefore, this paper brings together experiences of care-seeking and care-providing for recurrent vulvovaginal thrush.

## Method

A qualitative design was chosen to explore how recurrent vulvovaginal thrush is managed in primary care. Narrative and semi-structured interviews captured in-depth accounts through participants’ own terms. We approached analysis from a feminist perspective, recognising that gendered narratives shape how vulvovaginal symptoms are interpreted.

A patient representative group of seven women and one non-binary person (age range: 23–80 years) supported the design and dissemination of this study. We met at key points in the study and had five meetings to discuss recruitment materials, interview guides, initial analysis, and generating impact.

Recruitment for patient interviewees took place through community centres, support groups, and social media, as well as posters in GP offices, sexual health centres, and community pharmacies. Healthcare professionals were recruited via clinical research networks, newsletters, and posters in clinic settings. We purposively sampled both groups for maximum variation across demographics (ethnicity, gender, age) and experiences (See Tables 1 and 2).^13^ We included individuals who had confirmed recurrent thrush, and those who self-identified. Informed consent was obtained from all participants before interviews.

**Table 1.** 'Characteristics of patient sample

Name^a^	Age, years	Gender^b^	Ethnicity^b^	Duration of thrush, years
KJ	42	Gender fluid	White British	>10
Ayesha	25	Woman	Pakistani	4
Teddy	21	Non-binary	White British	3
Nancy	37	Woman	White British	6
Emily	32	Woman	White British	10, resolved
Aditi	22	Woman	Indian	2, resolved
Sai	24	Woman	Indian	4
Etta	50	Non-binary	White British	6
Nysha	40	Woman	Black British	>10
Beth	25	Woman	White British	10
Laura	42	Woman	White British	>10
Imani	35	Woman	Black African	2, resolved
Jody	26	Woman	White British	5
Elliott	30	Trans	White British	2
Billie	25	Woman	White British	7
Joy	43	Woman	White British	9 months
Sasha	34	Woman	Black African	1
Zoya	33	Woman	Pakistani	3
Kayla	42	Woman	White British	6
Lydia	26	Woman	White British	1
Leah	26	Woman	White British	10
Anna	34	Woman	Mixed race	10, resolved
Georgia	27	Woman	White British	4
Emma	41	Woman	White British	>10, resolved
Marie	60	Woman	White British	5
Harry	25	Woman	White British	2
Julia	36	Woman	White British	>10
Hannah	31	Woman	White British/Polish	2
Chloe	30	Woman	White British	>10
Imogen	29	Woman	White British	>10, resolved
Rowan	24	Woman	White British	2, resolved
Sarah	35	Woman	White British	10

^a^Name chosen by participant. ^b^Gender and ethnicity were self-identified.

**Table 2.** Characteristics of healthcare professional sample

Name^a^	Role	Age, years	Gender^b^	Ethnicity^b^	Experience, years	Location
Dr A	GP partner	52	Woman	White British	25	South East
Dr B	Foundation year doctor	24	Woman	White British	2	South West
Dr F	GP	35	Woman	White British	7	South East
Dr H	GP	32	Woman	British Pakistani	2	East Midlands
Ms I	Physician associate	35	Woman	Indian	10	Yorkshire
Dr K	GP	51	Woman	White British	13	South East
Dr M	GP partner	33	Woman	White Irish	10	North West
Dr P	GP partner	44	Woman	White British	11	London
Dr Q	GP	53	Woman	White British	22	North West
Dr R	GP	37	Woman	White British	6	West Midlands
Dr T	GP	36	Man	White British	3	North West
Dr U	GP	33	Man	Black African	8	East Midlands
Dr W	GP	35	Woman	British Chinese	5	North West
Dr X	GP community gynaecology	40	Woman	White British	12	South East
Ms C	Registered nurse	31	Woman	White British	5	North West
Dr D	SH consultant	38	Man	White British	13	London
Dr E	SH doctor	34	Man	Asian British	4	London
Dr G	SH doctor	43	Man	Asian	15	London
Dr J	GUM consultant	59	Woman	White Other	29	London
Dr L	SH doctor	32	Woman	Black/White	3.5	London
Dr N	SH consultant	42	Woman	White Irish	15	North West
Dr O	SH consultant	47	Woman	White British	16	South West
Dr S	SH doctor, GP registrar	29	Woman	Asian/White	2.5	South West
Dr V	SH doctor	38	Woman	White British	9	North West
Dr Y	SH associate specialist	59	Woman	White British	15	South East

a Names are pseudonyms. ^b^Gender and ethnicity were self-identified. GUM = genitourinary medicine. SH= sexual health.

Patient interviews began with a narrative prompt: participants were invited to describe what happened from the moment they first suspected something was wrong.^14^ This was followed by semi-structured questions to further explore key topics.^15^ Interviews were conducted mainly via video call, but also by phone, or in person, based on participant preference.

For healthcare professional interviews, we used a vignette-based approach. Participants were presented with a fictional clinical scenario, developed with input from patients and clinicians, and asked to reflect on how they might respond.^16^ This method was intended to elicit detailed responses without requiring discussion of specific patients.^17,18^ Semi-structured questions were laid out in a topic guide between layers of the vignette. Prompts encouraged reflection based on real experiences rather than purely hypothetical reasoning.

All interviews were audio-recorded, transcribed verbatim, de-identified, and analysed in NVivo12. Analysis was initially conducted separately for patient and clinician data, and then brought together. The first author carried out interviews, reflexive thematic analysis, and initial theme generation, which were then reviewed in discussion with the wider study team. Then, all codes relevant to healthcare encounters and care pathways were exported and analysed together using the one sheet of paper (OSOP) method.^15^

To deepen our analysis, we then consulted existing models of patient routes to consultation. Recognising that various models existed, but were limited to disciplinary silos, Wyke *et al* (2013) developed the integrated symptom-response framework to highlight how the self, social interaction, cultural expectations, and social structures overlap to influence symptom interpretation, evaluation, and action.^19^ We used this model to inform our analysis as it foregrounds how changes in knowledge, embodied state, and emotions are connected, changeable, and simultaneous.^19^

An online resource with further analyses and extracts from patient interviews can be found at: https://hexi.ox.ac.uk/Recurrent-Vulvovaginal-Thrush/overview .

## Results

Interviews were conducted with 32 patients self-identifying with recurrent thrush (May 2022–June 2023) and 25 primary care or sexual health professionals (May–July 2024). Patient interviews lasted 45–90 min; clinician interviews 30–60 min. Demographic information can be found in Supplementary Boxes S1 and S2. Pseudonyms were used throughout: patients were invited to use an alias or their first names; while healthcare professionals were labelled with an initial and their job role.

Below we present three key themes illustrating areas where the needs of people with recurrent vulvovaginal thrush diverged from acute cases:

- navigating disjointed health services;
- recognising and responding to recurrence; and
- building ongoing healthcare relationships.

### Navigating disjointed health services

Patient and healthcare professionals agreed that acute, transient, and one-off cases of thrush could be self-managed effectively through pharmacy care. Although there was recognition that pathways for recurrence needed to be different from those based on acute symptoms, transitioning between these and recognising when to do so was challenging.

Participants held various views on where recurrent thrush could be best managed and felt that signposting led patents in multiple, sometimes diverging, directions. Identifying, understanding, and knowing which services to see, and how often, was described as opaque and difficult to navigate. Patients expressed feeling caught in a void between services without a clear pathway.

*‘The thing is with the chemist, if they say to you, “Oh, how many episodes of thrush have you had in* [the] *past so many months?” and I’m honest, they won’t give* [medication] *to me, they’ll say, “You’ve got to go to your GP”, well then, your GP will say to go to the pharmacy*.’ (Leah, 26 years, woman, patient)

*‘Every time that* [thrush] *came back, when I went to the pharmacy, or made a GP appointment, I was like is this the right level of concern?’* (Rowan, 24 years, woman, patient)

Healthcare professionals across general practice and sexual health clinics held differing opinions about which services were best suited to manage recurrent thrush. These viewpoints were influenced by regional differences in commissioning, resources, and accessibility of services. In some centres, sexual health services were able, allowed, and financed to provide care for recurrent thrush, with some offering specialist vulval clinics within genitourinary medicine (GUM) departments. In other regions, general practice was seen as the most appropriate avenue.

*‘The biggest challenge is that patients don’t know where to go and there is nowhere for them to go, properly.* […] [At our sexual health clinic] *we are not supposed to see these patients. We are supposed to say, go back to your GP.* [… ] *I’ve masses of respect for GPs, but, sexual health doctors are trained in management of this sort of condition. There isn’t any joined up commissioning.’* (Dr O, 47 years, woman, Sexual health [SH] consultant)

Gaps in information sharing and communication across services were identified as an additional barrier to recognising a transition from acute episodes to recurrent thrush:

*‘You aren't necessarily seeing the same GP each time, maybe not even a GP, you could go to the pharmacy, a nurse practitioner, maybe* [a] *sexual health* [clinic]*. We can't see sexual health notes. From general practice, we have this blindside*.’ (Dr X, 40 years, woman, GP working in community gynaecology)

Patients with underlying conditions that can exacerbate recurrent thrush, such as HIV or diabetes, also faced challenges with knowing where to access collaborative care. For instance, Joy (43 years, woman, patient) said that her HIV team did not know much about recurrent thrush, and Teddy expressed confusion over what notes their diabetes team could access:

*‘I assumed all my records would be together. I'm starting to think they're not.’* (Teddy, 21 years, non-binary, patient)

To address these challenges, some healthcare professionals tried to overcome silos and increase communication within and between sectors using written plans.

*‘I’m a big believer in writing a plan so that other* [clinicians] *know what you were thinking, because I'd hate* [the patient] *to come back and then what I'd said was going to happen doesn't happen*.’ (Dr Y, 59 years, woman, SH associate specialist)

Dr S (SH doctor, GP registrar) explained shared plans help create a ‘two-way system’ between sexual health clinicians and primary care professionals. Patients were also able to ask for their notes to be shared with them and bring those to their appointments to participate in collaborative plans. Within an overstretched health system, clinicians recognised that patients play an active role in coordinating and navigating their care journeys:

*‘There’s a bit of fragmentation* […] *The person now that most notices the pattern, is the person experiencing the symptoms. If anyone in the system knows what’s going on, it’ll be the person. They’re the one unifying feature*.’ (Dr A, 52 years, woman, GP partner)

Recurrent thrush was viewed as typically manageable within primary care or sexual health services, but unresponsive or evolving symptoms prompted consideration about referrals to secondary care. Some clinicians contacted gynaecology for advice or referrals, but others expressed hesitancy to place patients on long wait lists for gynaecology when the treatment options would not differ from those available in primary care. When other conditions were suspected with similar symptoms (for instance lichen sclerosus) referrals to vulval dermatology were seen as more appropriate.

Calls were made for more collaboration and clearer guidance within primary care regarding who should manage recurrent thrush (and in what order and which cases).

*‘The expertise already exists, it doesn't have to be in a women’s health hub, but a clear system that everybody understands would be really helpful*.’ (Dr O, 47 years, woman, SH consultant)

### Recognising and responding to recurrence

When making decisions about how, when, and where to engage with health care, patients drew from past healthcare experiences as to whether they had felt the issue was (or would be) treated as ‘serious’. For patients, being taken ‘seriously’ in these cases often meant feeling that recurrent thrush was recognised as distinct from one-off cases, and necessitated a transition to different management and support:

*‘I think the difference is how it impacts your life. Because if I had* [thrush] *once or twice, it would be uncomfortable, but when you get it recurrently, it’s a completely different condition. I think they should almost be treated completely separately and not related.’* (Harry, 25 years, woman, patient)

*‘I think because* [thrush] *is common,* [recurrent thrush] *gets dismissed, it’s not seen as a serious health issue. I think we need to separate out those very common episodes from once it starts getting to be more recurrent.’* (Dr Y, 59 years, woman, SH associate specialist)

Although all cases of recurrent thrush will begin with a first episode, recognising the transition between one-off to ongoing helped recognise the distinct psychological impact and management challenges. A sexual health doctor reflected that while acute thrush was ‘quite easy’ to manage, recurrent thrush ‘needs a bit more thinking about’ (Dr E, SH doctor). A GP thought the impact on people’s body image and relationships made recurrent thrush ‘really complex for everybody’ (Dr K, GP).

Further tensions could arise when clinicians expressed caution around their language to avoid alarming patients, whereas patients worried that this meant their concerns were not taken seriously. For example, some clinicians weighed up referring patients to ‘complex’ sexual health or GUM clinic that could offer help for recurrent thrush, but were perceived as possibly causing alarm:

*‘They’re then hearing the term that we have for the clinic, which is the “complex clinic”, that’s probably a bit daunting hearing that term.* […] *“But it’s not a complex... is this a complex issue?” so I imagine it can be quite confusing.* (Dr F, 35 years, woman, GP)

Feeling taken ‘seriously’ also related to larger social narratives that framed recurrent thrush as something to tolerate. These accounts were rooted in participants’ own experiences, but also drew on social conversations about gender health inequities. Recurrent thrush was framed as a ‘woman’s issue’, which had an impact on how participants (including cis-women, trans, non-binary, and gender fluid people) understood the condition. Teddy who is non-binary, and KJ who is gender fluid, said people with vaginas were told to expect discomfort, ‘tough it out’, or ‘soldier on’. Many clinicians raised concern over these gendered narratives, as one GP said that recurrent thrush was ‘wrongly minimised because of the perception that “oh it’s a women’s issue so it’s a minor thing”.’

When a clinician was able to communicate that recurrent thrush required a distinct approach, plan, and validation of its impact, patients felt more able to return to seek care:

*‘I remember doctors not seeming that concerned or interested, until I saw this amazing doctor. He was really listening and took it really seriously. He was like, “This is really serious, I'm so glad you've ended up here”.’* (Anna, 34 years, woman, patient)

Patients also felt recurrence was recognised when they were prescribed longer-term treatment plans instead of advice and medication suited for a one-off thrush episode:

*‘I had a really positive experience initially with the GP who took it quite seriously and gave me 6 months’ worth of capsules to take once a week.’* (Julia,36 years, woman, patient)

### Building ongoing healthcare relationships

Recurrent thrush necessitated repeated care, which meant that ongoing and enduring relationships with healthcare professionals were important. An ongoing relationship with a healthcare professional enabled monitoring progress and changes over time. Patients found it difficult to see a different clinician each time and re-explain their experience:

*‘I’m lucky that I have a very good relationship with my GP. Now that this has happened in the past, I am kind of confident that I’d be listened to and it wouldn’t be minimised.’* (Rowan, 24 years, woman, patient)

Managing expectations was also central to building ongoing relationships. During early consultations, patients anticipated a ‘quick fix’, or ‘cure’. Over time, they transitioned to see recurrent thrush as requiring ongoing management. By presenting a plan for follow-up, patients felt heard and healthcare professionals were able to gain trust towards managing this condition:

*‘I say, “I am going to help you, but you need to persist with me …” I say you must come back to me at this time and we will review it again, and this will be the plan. Then they’ll trust you. Even if they have a blip, they’ll still trust you. The plan is sound.’* (Dr Y, 59 years, woman, SH associate specialist)

Trust was a critical factor in patients feeling able to return to seek care. Patients and clinicians agreed that trusting relationships were shaken through small acts, such as missing follow-up phone calls, losing samples, and not planning next steps.

Some healthcare professionals noted that saying “please come back” was often not enough when patients were unsure whether recurrent thrush warranted further attention or whether they may get lost in a disjointed system. A sexual health doctor (Dr L, SH doctor) tried to establish trust with patients by saying: ‘you are not on your own’ and approach longer-term treatment as ‘we’re doing this together, teamwork’. Dr E (SH) explained their approach to continuity of care:

*‘*[In] *sexual health services, if they see me one week, I can easily put them into my clinic list two weeks later, and three weeks later, a month later.’* (Dr E, 34 years, man, SH doctor)

However, other clinicians did not (or could not) routinely offer follow-up appointments, rather encouraging patients to return if symptoms did not resolve:

*‘I don’t think I’ve ever seen a thrush patient and said, “I think we need to see you again in a few weeks to see how you’re getting on – let me book you in.” I’ve probably just said, “If things aren’t improving, or your symptoms change, please come back” and left the ball in their court*.’ (Ms I, 35 years, woman, physician associate)

Some patients found that learning more about health services and systems (whether through repeat appointments, support groups, social media, or research) helped them gain confidence in self-advocating. This included feeling more able to ask questions and request additional appointments. Self-advocacy was framed as enabling conversations around collaborative care:

*‘It’s really hard to sit in front of a doctor and go, “I don't agree with you”, but it might make them question it and go, “oh right, OK, if you don’t agree, how can we work together to sort it out?”.’* (Billie, 25 years, woman, patient)

However, some patients reported frustration growing over time or feeling unable to self-advocate, and clinicians recognised these limitations:

*‘There’s loads of barriers and taboos around women’s health. Some they’re very determined they will keep going back to the doctor, those patients I’m not worried about, but the patients who have language barriers, or who have access issues, those ones I’m concerned about*.’ (Dr F, 35 years, woman, GP)

Building ongoing healthcare relationships was not always straightforward or linear, but once established provided a valued space for collaborative care and informational continuity.

## Discussion

### Summary

This paper examined the care pathways people described when seeking and providing help for recurrent vulvovaginal thrush, and the experiences they amassed along the way. Current approaches fail to capture how recurrent thrush experiences evolve to become different for the entire care-seeking pathway, which is marked by repetition, reversal, and retraction. Recurrent thrush may begin with an acute episode, but is not equivalent to acute episodes on repeat. Instead, it requires distinct attention. Help for recurrent thrush requires patients to remain engaged with healthcare systems. Recurrent thrush differs from acute cases of thrush not because of symptom presentation, but in its repetitive nature — with implications for services, care pathways and healthcare relationships. However, knowing how and when to transition between the pathways for acute or recurrent cases of thrush, and having systems that enable or support this transition remains difficult.

Our study revealed the challenges of treating a condition that straddles the line between commonality and complexity. Patients and clinicians were attempting to navigate constrained and fractured care structures that both parties can either tolerate or work together to improve.

### Strengths and limitations

This paper brought together patient and clinician voices to better understand perspectives from both groups. Qualitative methods allowed in-depth exploration into experiences, expectations, and perceptions of giving and receiving care. Interviewees were recruited through varied avenues and represented different ages, ethnicities, gender diversity, and socioeconomic statuses. The people who participated represented those willing to speak about their condition at interview, but some shared that it was their first time talking openly.

All interviews were conducted by one researcher with lived experience of recurrent vulval pain, which may have influenced data interpretation. To mitigate this, ongoing discussions with the wider research team and reflexive journaling were used to examine assumptions and enhance data interpretation.

The patients and clinicians represented in this study may be those who are particularly engaged with vulvovaginal health. This allowed thoughtful discussion, but may mean some perspectives are missing. Nonetheless, the fact that these healthcare professionals and patients highlighted challenges with management may mean others are also facing similar situations.

As with all qualitative studies, the findings represent situated interpretations within a particular social and clinical context.

### Comparison with existing literature

Existing models for help-seeking for recurrent vulvovaginal symptoms are oversimplified and struggle to capture the difficulty of transitioning from acute approaches to providing long-term care for those with recurrent cases. Attempts to map recurrent thrush have been presented as a series of discrete steps with a linear path, sequential order, and single direction.^11,12^ The models appear misleadingly stagnant, straightforward, and unchanged as people move through them. The lines do not overlap or intersect to acknowledge how patients are navigating multiple care systems and considerations simultaneously. Instead, we found care pathways were described as ‘fractured’ and ‘fragmented’ with patients ‘slipping through the cracks’. Our findings highlight that help-seeking and help-providing for recurrent thrush is neither linear nor unidirectional, but instead involves capturing transitions and multiple pathways that open up or close down options for both patients and clinicians.

Further, existing models present the option to return to the start and re-enter the path in a circular fashion.^11,12^ Our findings demonstrate that care-seeking is not a loop, but instead leads to new paths filled with different outcomes, experiences, and expectations. Patients and healthcare professionals are not moving through these systems unchanged, but instead amassing experiences during and between healthcare encounters.

Our findings reinforce previous publications reporting women and gender diverse patients feeling dismissed in health care.^4,6–8,20,21^ However, whereas past work positions care providers as uninterested in recurrent thrush due to gender inequities, we instead found healthcare professionals (mainly women themselves) acknowledging, and often challenging, the same gendered narratives as patients.

**Figure 1.**
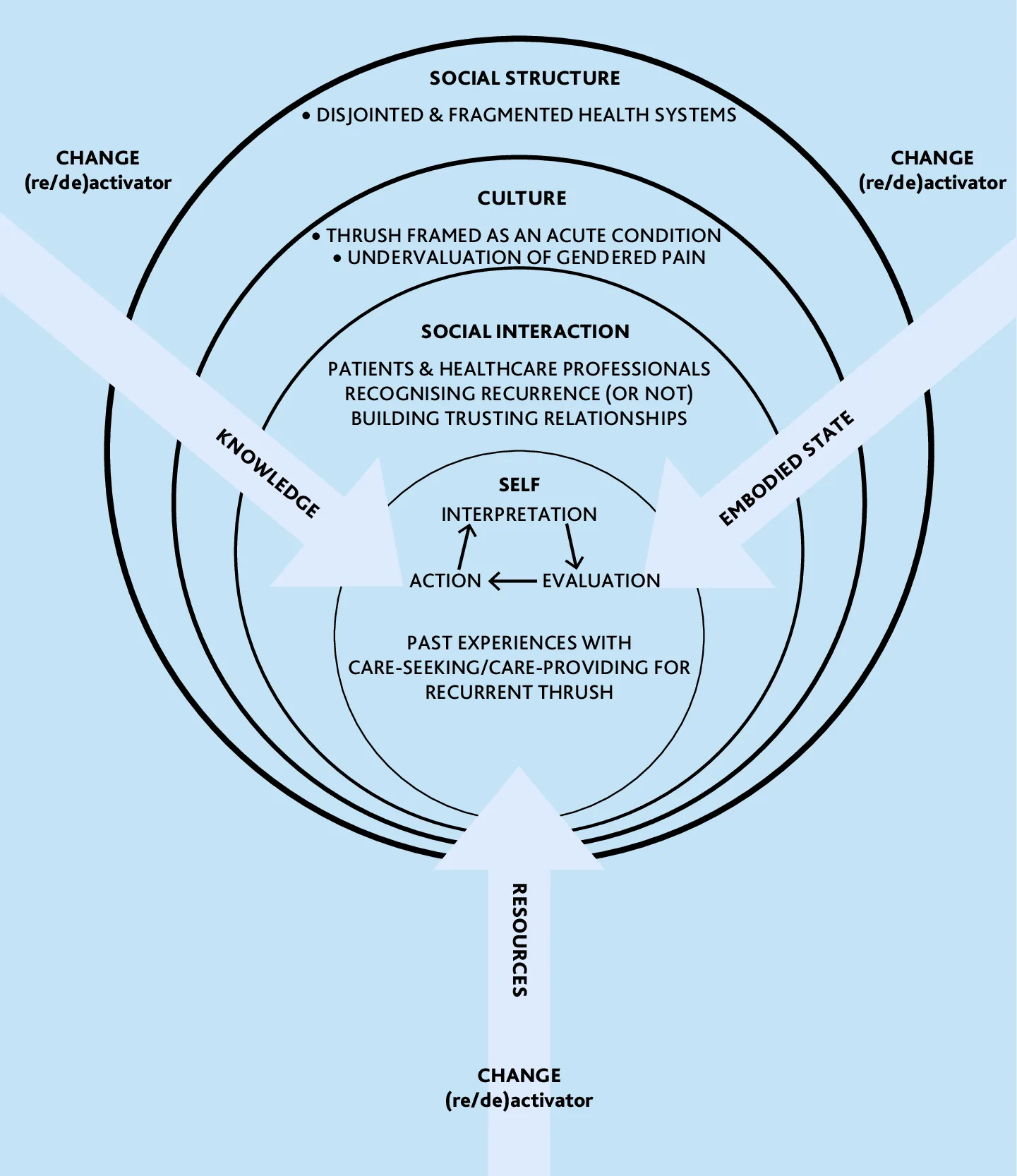
The integrated symptom-response framework as it applies to recurrent vulvovaginal thrush care pathways (adapted from Wyke *et al*).^19^

The integrated symptom-response framework presents an alternative model that helps capture the complex and multilayered nature of participant healthcare journeys by highlighting the changes in needs, experience, and knowledge accumulation (see Figure 1).^19^ Although this model has focused on care-seeking, our study demonstrates that healthcare professionals did not operate in a separate schema detached from patients, but were instead embedded within the same social structure and culture that influenced their clinical interactions.

As experiences — of recurrence and of help-seeking/providing — accumulated over time, there were implications around the limits of care primarily structured around one-off episodes. This accumulation was not limited to factual knowledge, but to the embodied, emotional, and experiential elements that people picked up along their care journeys. By invoking the process of ‘accumulation’, we speak to the process in which summative experiences are amassed over time, into a complex amalgamation of everything that has come before, whether lived, anticipated, or absorbed from others. It goes beyond adding together multiple one-off experiences, and instead speaks to the experience of recurrence as exceeding or changing in nature the sum of its parts.

Wider literature on similar conditions such as recurrent bacterial vaginosis and recurrent urinary tract infections have highlighted patient dissatisfaction with clinical management, frustration with recurrence, and perceived dismissal.^22,23^ Across these cases, recurrence plays a key role in generating uncertainty around a condition that is typically seen as one-off, acute, minor, and non-serious. The overlap suggests that our accumulative approach discussed here may have broader relevance for understanding other recurrent conditions.

### Implications for research and practice

Recurrent thrush requires distinct attention, care pathways, and relationships that transition away from those offered for acute, transient, and one-off episodes. Patients found that they required multiple appointments and follow-up care to receive an effective management plan. Primary care professionals are well positioned to help patients with recurrent thrush, but structural, systemic, and social barriers are making this more difficult.

Current healthcare systems are overburdened, and each service faces their own local conditions and challenges. Therefore, any recommendations must be considered in this context (Box 1).

Box 1. **Recommendations for improving recurrent vulvovaginal thrush care experiences**

- **Prompt patients about recurrence**: ask whether this is something that happens repeatedly and if patients have sought care elsewhere
- **Support primary care networks to identify recurrence in their patient population:** through agreed coding of episodes and suggestions for audit approaches. Record episodes of recurrence carefully in notes to help patients get on a pathway towards longer-term care
- **Prioritise continuity of care and relationship building:** write down and verbally explain management plans involving follow-up and next steps for patients that encourage them to remain involved with health care
- **Facilitate opportunities for informational continuity:** identify what information can be shared within and between health services (through patient-approved note-sharing, writing letters between departments, and working across sexual health and general practice for further community management)
- **Consider the different routes of presentation of acute thrush across community settings**: consider what can be done in these encounters to guide patients regarding the most appropriate actions in case of a recurrence. Highlight the next steps and signpost to available local services if a recurrence occurs
- **Understand the widespread impact of recurrent thrush and its accumulative impacts:** recognise that recurrent thrush is a distinct experience from acute episodes and has different impacts and considerations

In conclusion, this paper examined experiences of recurrent thrush in their own right, not as subsections, footnotes, or outliers of acute experiences. In doing so, we found there was a shared belief that recurrent vulvovaginal thrush can be managed effectively in primary care but requires distinct attention. Recurrent conditions are not the same as acute episodes on repeat, but instead distinct experiences with unique healthcare needs that warrant further attention. Using the concept of accumulation, we highlighted how recurrent conditions become larger than the sum of their episodes. These care journeys became accumulative and expansive as people’s choices relate to past experiences as well as larger social narratives and anticipated destinations.
